# Novel Aspects of Extracellular Vesicles as Mediators of Cancer-Associated Thrombosis

**DOI:** 10.3390/cells8070716

**Published:** 2019-07-13

**Authors:** Vitor H. Almeida, Araci M. R. Rondon, Tainá Gomes, Robson Q. Monteiro

**Affiliations:** Institute of Medical Biochemistry Leopoldo de Meis, Federal University of Rio de Janeiro, 21941-160 Rio de Janeiro, Brazil

**Keywords:** extracellular vesicles, microvesicles, exosomes, cancer, thrombosis, tissue factor, platelets, neutrophils, neutrophil extracellular traps, polyphosphate

## Abstract

The establishment of prothrombotic states during cancer progression is well reported but the precise mechanisms underlying this process remain elusive. A number of studies have implicated the presence of the clotting initiator protein, tissue factor (TF), in circulating tumor-derived extracellular vesicles (EVs) with thrombotic manifestations in certain cancer types. Tumor cells, as well as tumor-derived EVs, may activate and promote platelet aggregation by TF-dependent and independent pathways. Cancer cells and their secreted EVs may also facilitate the formation of neutrophil extracellular traps (NETs), which may contribute to thrombus development. Alternatively, the presence of polyphosphate (polyP) in tumor-derived EVs may promote thrombosis through a TF-independent route. We conclude that the contribution of EVs to cancer coagulopathy is quite complex, in which one or more mechanisms may take place in a certain cancer type. In this context, strategies that could attenuate the crosstalk between the proposed pro-hemostatic routes could potentially reduce cancer-associated thrombosis.

## 1. Introduction

The occurrence of prothrombotic events such as deep vein thrombosis, venous thromboembolism (VTE) and stroke may greatly vary across different cancer types. It is estimated that one out of five cancer patients will develop this co-morbidity during the tumor progression. Indeed, there is a significantly increased risk of thromboembolism in cancer patients as compared to the general population [1,2]. This correlation, independently noted by Dr. Jean-Baptiste Bouillaud and Dr. Armand Trousseau in the 19th century, has been well reported [3,4]. Clinical risk factors for thrombotic complications include a wide range of factors such as patient-related, cancer-related and treatment-related risk factors [5,6].

Cancer-associated thrombosis is a multi-factorial process that has been associated with several mechanisms [7,8]. The involvement of extracellular vesicles (EVs) in this process has been proposed. According to the 2018 guideline of the International Society for Extracellular Vesicles (ISEV), EVs can be defined as “the generic term for naturally released particles from the cell that are delimited by a lipid bilayer and cannot replicate” [9,10,11]. Whenever possible, the nomenclature of the EVs follows the updated guidelines of the ISEV to ensure correct scientific disclosure [10]. EVs have been extensively associated with intercellular communication in both physiological and pathological situations, including thrombosis and cancer. Tumor-secreted EVs are able to achieve systemic circulation in animal models [12,13], as well as in patients [14,15,16]. These vesicles carry genetic material, as well as other macromolecules derived from the tumor, modulating biological responses in the host [17]. The two major populations of EVs released by tumor cells are microvesicles and exosomes [9,10,18]. Microvesicles (ectosomes/ microparticles) are lipid bilayer-enclosed sacs (100 to 1000 nm of diameter) released from the plasma membrane to the extracellular milieu [9,10,18]. Under physiological conditions, several cell types are able to release microvesicles, however, the malignant transformation stimulates the release of these EVs [12,19]. Exosomes differ from microvesicles by their size (30 to 100 nm), formation process (endosomal origin) and protein content [9,10,18]. As seen with tumor cells, cancer cell-derived EVs may expose procoagulant phospholipids such as phosphatidylserine, which constitute a suitable platform for the assembly of the blood coagulation complexes [20,21]. However, other EV-related prothrombotic mechanisms have been recently proposed and will be discussed here.

## 2. Tissue Factor-Bearing EVs in Cancer-Associated Thrombosis

Tissue factor (TF/*F3* gene) is a 47-kDa transmembrane protein that initiates the extrinsic pathway of coagulation upon binding to circulating factor VII/VIIa (FVII/ FVIIa/ *F7* gene). The binary TF/FVIIa complex further activates FIX (*F9* gene) and FX (*F10* gene) into their active forms leading to thrombin (*F2* gene) production, which culminates with fibrin generation [22]. The presence of TF in blood as an exposed component of EVs derived from vascular cells, and later on tumor cells, rapidly leads to the proposal that the TF plasma levels would directly reflect the prothrombotic state of cancer patients. Most of the studies were initially performed with EVs in the microvesicle-size range. Indeed, several studies demonstrated that EVs isolated from cultured cells expose TF to similar extents as observed in the producing cells [23]. Malignant transformation appears as a major trigger not only for increased cellular vesiculation, but also for coagulopathy [24,25]. Indeed, cancer driver mutations and the overactivation of signaling pathways increase the expression of TF in tumor cells [26,27,28], as well as trigger the emission of TF-bearing EVs [29]. Other cancer-related phenomena, such as the epithelial-mesenchymal transition (EMT), may also promote the release of EVs containing TF [29].

Mouse models have been widely used to study mechanisms involved in cancer-associated thrombosis, although it is known that clotting parameters may vary across different animal species and mouse strains [30,31,32]. In this context, most of the studies that evaluated the effects of TF+ EVs on cancer-associated thrombosis employing mouse models were performed using pancreatic cancer cells [31,33], as this cancer type presents the highest incidence of VTE in cancer patients [1]. Mouse models employing either the orthotopic or the ectopic pancreatic cancer cell implantation have consistently demonstrated the establishment of prothrombotic states [13,34,35,36,37,38]. EVs derived from the murine Panc02 cancer cell line promote increased thrombus formation (induced by FeCl_3_ or laser-induced injury) in a TF-dependent pathway and accumulate in the injury area [35]. Interestingly, a recent report showed that the intravenous administration of TF+ EVs derived from pancreatic cancer cells induces deep vein thrombosis in mice. In this model, TF on pancreatic tumor-derived EVs must cooperate with host TF to evoke a prothrombotic state in the animals [39].

Additional studies employing Lewis lung carcinoma and melanoma cell lines have been performed using mouse models. These studies have demonstrated the establishment of TF+ EV-dependent prothrombotic states [12,35]. Our group showed that melanoma-derived EVs display greater procoagulant activity and higher levels of TF as compared to melanocyte-derived EVs. By using an arterial thrombosis model, we observed that the intravenous administration of melanoma EVs, unlike melanocyte EVs, accelerate thrombus formation in naïve mice. This effect was dependent on the presence of TF since active site-blocked FVIIa (which works as an antagonist of FVIIa/TF complex) completely abolished the thrombogenicity of these vesicles [12]. Remarkably, tumor-bearing mice exhibit a high level of TF+ melanoma-derived EVs in plasma [12].

Several studies have tried to correlate the presence of circulating TF+ EVs and the occurrence of VTE in cancer patients. It is important to emphasize that different parameters have been used in these studies, including the evaluation of TF antigen levels (flow cytometry, impedance-based flow cytometry or ELISA) and/or the evaluation of TF activity (FXa generation or fibrin generation test) [40]. These parameters have distinct sensibilities and consequently diverse outcomes. In this context, TF activity assays have been considered as a more attractive option to evaluate this association, as they exhibit higher sensibility.

Increased TF antigen levels were initially identified in EVs isolated from plasma samples derived from advanced colorectal cancer patients [14]. Further studies demonstrated the elevation of circulating TF+ EVs in other tumor types including pancreatic, lung, ovarian, colorectal, and breast cancers [15,41,42]. A positive correlation between TF+ EVs and VTE in pancreatic cancer patients was demonstrated in other studies [43,44,45]. On the other hand, contradictory results are reported for the positive association between TF+ EVs and VTE occurrence in breast cancer patients [43,46]. Other findings failed in revealing an association between TF+ EVs with VTE in soft tissue sarcoma patients [47], as well as in non-Hodgkin lymphoma, colorectal, breast, stomach, lung and pancreatic cancers [48]. Additional reports did not show an association between the TF activity levels in EVs and VTE in multiple myeloma [49], ovarian carcinoma [50,51], small cell lung cancer [52], and gastric, colorectal and brain tumors [53]. However, in multiple myeloma patients with VTE after chemotherapy, it was observed that higher levels of TF activity occured in comparison with patients that did not develop thrombosis [49]. The compilation of the studies that evaluated TF+ EVs and occurrence of VTE is shown in Table 1.

Conflicting outcomes from studies that tried to correlate TF + EVs in plasma from cancer patients and occurrence of VTE are possibly due to the use of different techniques for TF measurement (antigen or activity), EVs purification methods, the sensibility of antibodies and distinct assays. In the last years, a great effort from ISEV to establish guidelines for studies with EVs has been done [10]. Additionally, some groups are developing new activity assays for TF+ detection in plasma-derived EVs [54]. These efforts are crucial for a better understanding of the role of TF+ EVs and cancer-associated thrombosis.

## 3. Platelets in Cancer-Associated Thrombosis

Platelets are fragments (2–4 μm in diameter) extruded from bone marrow megakaryocytes and released into the bloodstream. Under physiological conditions, the platelet concentration in humans ranged from 150 to 350 × 10^3^/µL. Platelets are key players in hemostasis since they detect endothelial injury through several receptors [55]. In the basal state, platelets circulate without forming adhesions with the endothelium. In the presence of a vascular lesion, the platelet glycoproteins GPVI and GPIb-V-IX bind to the collagen from the subendothelial matrix and to the von Willebrand factor, respectively. These interactions mediate platelet activation and adhesion to the site of vascular damage. Upon activation, platelets change their morphology, degranulate and release agonists, such as adenosine diphosphate (ADP) and thromboxane A2 (TXA2). These events contribute to further platelet aggregation [55,56]. Cancer may influence the platelet count, physiology and activation state.

One of the risk factors for VTE is the elevated platelet count [5,57,58]. The Vienna Cancer and Thrombosis Study (CATS) showed that thrombocytosis was an independent risk factor for VTE in cancer patients. Patients with platelet count ≥ 443 × 10^3^/μL were 3.5 times more likely to develop VTE as compared to the group with counts below the designated cut-off [59]. Other studies have also shown that patients with malignant neoplasms, including gastrointestinal, endometrial, pancreatic, and colorectal cancers, develop thrombocytosis [60,61,62,63]. The mechanisms involved in tumor-induced thrombocytosis are still not fully understood. One of the possible mechanisms described in the literature is that tumor cells may produce and secrete humoral factors that influence platelet production through an endocrine activity in the megakaryopoiesis. These factors comprise vascular endothelial growth factor (VEGF), granulocyte-macrophage colony-stimulating factor (GM-CSF), granulocyte colony-stimulating factor (G-CSF), interleukin-6 (IL-6), thrombopoietin (TPO), and basic fibroblast growth factor (b-FGF) [64,65,66,67].

An important study, which included 619 patients with ovarian cancer, showed that thrombocytosis was associated with tumor progression and poor survival. In this study, paraneoplastic thrombocytosis was mediated by TPO and IL-6 [68]. Treatment with an anti-IL-6 neutralizing antibody reduced the number of platelets in tumor-bearing mice as well as in patients with ovarian cancer. In addition, IL-6 blockade in combination with paclitaxel had a synergistic effect on reducing tumor growth in murine models of epithelial ovarian cancer [68]. The use of an antiplatelet antibody significantly reduced platelet counts as well as tumor growth in vivo, inducing tumor necrosis [68]. These results suggest positive crosstalk between tumor cells and platelets, which contributes to the aggressive behavior of ovarian cancer. Cancer-associated thrombocytosis also has a negative prognostic value in other types of cancer [69,70].

In addition to thrombocytosis, tumor cells may activate platelets by direct interaction or indirectly via EVs or secreted soluble factors [71]. Indeed, it was shown that circulating platelets of cancer patients express high levels of P-selectin, a marker of platelet activation [72]. Platelet function largely depends on integrin signaling, including α2β1, α5β1, α6β1, αIIbβ3 and αvβ3 [73]. Tumor cell-induced platelet aggregation can occur through the binding of the platelet αIIbβ3 integrin to the αvβ3 integrin of tumor cells via proteins containing the RGD motif, such as fibronectin and fibrinogen [74,75]. Platelet α6β1 integrin has also been shown to be capable of binding to tumor cells, inducing platelet activation [76].

Tumor cells can activate platelets through the production/secretion of soluble factors, such as ADP, TXA2, thrombin, cathepsins and matrix metalloproteinases (MMPs) [71]. Platelets express two ADP-activated G protein-coupled receptors (GPCRs), P2Y1 and P2Y12. Tumor cells have been described to release ADP in the extracellular environment, thus contributing to platelet activation/aggregation [77,78]. TXA2 is a platelet agonist, and its receptor is also a GPCR expressed on platelets. Several neoplasms, including lung, bladder, colorectal, prostate and thyroid tumors, exhibit overexpression of the thromboxane synthase enzyme, which catalyzes the conversion of prostaglandin H2 into TXA2 [79,80,81,82,83], suggesting a possible mechanism of thromboxane secretion. Indeed, it has been shown that pharmacological inhibition of tumor-derived TXA2 synthesis suppresses platelet aggregation in vitro [84].

Thrombin is a serine protease that amplifies the coagulation cascade, activating several zymogens, and converting soluble fibrinogen into insoluble strands of fibrin, which stabilize the platelet plug in clot formation [22]. Thrombin is the most potent physiological activator of platelets. One class of GPCRs typically activated by proteolysis are the protease-activated receptors (PARs) and several proteases, in addition to thrombin, are capable of activating PARs [85]. It was shown that colon cancer cells may trigger human platelet activation in a manner that is dependent on the cancer cell TF expression, thrombin generation and activation of PAR-4 on platelets, inducing thrombus formation in vitro under flow [86]. Cathepsin K has also been shown to activate human platelets through a PAR-3 and -4 dependent mechanism [87]. Data from the literature show that cathepsin K is expressed in several types of cancer [88]. Other tumor-derived cathepsins were shown to aggregate platelets [89]. Recently, Sebastiano and colleagues have described a novel mechanism regulating platelet activation, which involves the binding of tumor-derived MMP-2 to the αIIbβ3 integrin of the platelet. Then, this metalloproteinase cleaves and activates PAR-1, inducing a pre-activated state in the platelet [90]. In addition to MMP-2, other metalloproteinases are involved in platelet biology [91].

In 2006, the C-type lectin-like receptor 2 (CLEC-2) was described as a novel platelet-receptor [92]. So far, the only ligand described for the CLEC-2 receptor is podoplanin. Podoplanin is a membrane glycoprotein expressed on the surface of several tumor cells [93,94]. In glioblastoma models, platelet aggregation in vitro appears to be dependent on podoplanin expression [95]. In addition, podoplanin overexpression in tumor samples from glioma patients was associated with intravascular platelet aggregates and increased risk of VTE [95]. A recent study has shown that intravenous inoculation of B16-F10 melanoma cells expressing podoplanin induced thrombus formation in the lungs of mice. The formation of the pulmonary thrombi was significantly reduced in mice that were previously treated with a monoclonal antibody against CLEC-2 [96]. Remarkably, tumor-derived microvesicles expressing podoplanin have been observed in the plasma of patients with pancreatic and colorectal cancer [97]. Therefore, the podoplanin-CLEC-2 axis may play an important role in platelet aggregation induced by tumor cells and, consequently, in the cancer-associated thrombosis.

We recently described that EVs (mostly in the exosome size-range) derived from MDA-MB-231 cells, an invasive and mesenchymal-like breast cancer cell line, showed higher TF protein levels than EVs derived from MCF-7, a non-aggressive and epithelial breast cancer cell line [98]. Accordingly, TF-dependent platelet aggregation was induced by MDA-MB-231 EVs, but not by MCF-7 EVs in platelet-rich plasma, since the coagulation cascade triggered by TF generates thrombin, a potent platelet agonist [98]. Interestingly, the interaction between MDA-MB-231 EVs and washed platelets (plasma free condition) also induced P-selectin exposure on the platelet surface, as well as platelet aggregation, suggesting a TF-independent mechanism [98]. Thomas and colleagues also showed that lung and pancreatic cancer cells-derived microvesicles aggregate platelets via a TF-dependent mechanism. Mice infused with these EVs exhibit reduced tail bleeding time and the establishment of a prothrombotic state [35]. In addition, Geddings and colleagues showed that microvesicles derived from pancreatic cancer cells mediate the activation/aggregation of human platelets in vitro in a manner dependent on TF and thrombin [13]. Moreover, injection of tumor EVs triggered platelet activation in vivo and increased venous thrombosis in a TF-dependent manner in mice [13]. They have also found that thrombosis induced by TF-expressing microvesicles was reduced in PAR-4 knockout mice or in clopidogrel-treated mice (an antiplatelet drug), corroborating the biological role of the platelets in this model [13]. These results suggest that the platelet activation mediated by TF-expressing microvesicles is necessary for the development of thrombosis in tumor-bearing mice in determined contexts.

**Figure 1:**
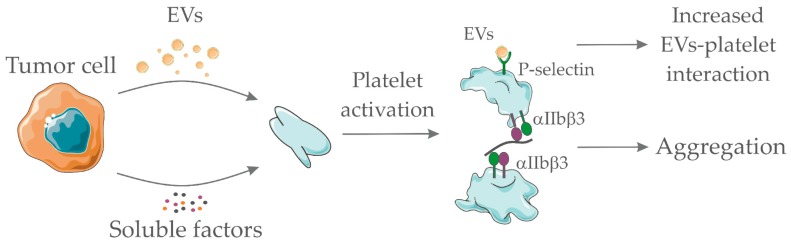
Pro-hemostatic interactions between tumor cell-derived soluble factors/extracellular vesicles (EVs) and platelets. Tumor-derived EVs and/or tumor-derived soluble factors (such as adenosine diphosphate, thromboxane A2, and others) interact with platelets promoting their activation. Platelet activation accounts for integrin αIIbβ3 exposure and further interaction with fibrinogen, thus enabling platelet aggregation. In addition, platelet activation promotes P-selectin exposure which serves as a ligand for P-selectin glycoprotein ligand 1 (PSGL1)-containing EVs. EVs interaction with platelets favor their accumulation at the site of thrombotic injury. Together, both processes favor thrombus formation. Servier Medical Art, https://smart.servier.com/, was used to create this figure, licensed under a Creative Commons Attribution 3.0 Unported License.

If on the one hand EVs derived from tumor cells activate platelets, on the other hand, it has been shown that activated platelets are fundamental for the accumulation of these vesicles at the site of thrombosis. This mechanism is dependent on the presence of P-selectin glycoprotein ligand 1 (PSGL-1) and integrins on the EVs membrane. Treatment with a P-selectin blocking antibody, RGD peptide (a competitive inhibitor of the integrin-ligand interactions), clopidogrel or depletion of circulating platelets prevented the accumulation of tumor-derived microvesicles at the site of injury, reducing the incidence of thrombotic events in the animal models [35,99]. Figure 1 depicts the proposed interaction between soluble factors/EVs derived from tumor cells and platelets.

## 4. Neutrophils and NETs in Cancer-Associated Thrombosis

Several recent reports have demonstrated that immune cells play a significant role in thrombus initiation and development [100]. In this context, as seen with platelets, elevated leukocyte/white blood cell (WBC) counts have been associated with the increased risk of thrombosis in cancer patients [57,101]. The Tromsø study reported that cancer patients presented with high WBC counts before the diagnosis had a 2.4-fold increased risk in developing VTE as compared to those that presented normal WBC counts. Interestingly, increased WBC counts were not predictive for VTE in a non-cancer population [102]. On the other hand, a recent study showed that persistent neutrophilia was associated with VTE in patients that did not present malignancies or infections [103]. In this study, persistent neutrophilia was defined as an elevated absolute neutrophil count in at least three blood exams with a minimum of two months apart. The association of VTE with the neutrophil to lymphocyte ratio (NLR), as well as the platelet to lymphocyte ratio (PLR), have been evaluated in cancer patients, with conflicting results. Ferroni and colleagues showed that patients with solid tumors had a 2-fold and 3-fold higher risk to develop VTE upon high NLR or PLR, respectively [104]. More recently, a study that evaluated 486 non-cancer patients with VTE showed that high NLR or PLR was not associated with an increased risk of VTE or cerebral vein thrombosis [105]. Another recent study with gastric cancer patients failed in showing NLR or PLR as predictors for VTE [106].

Among immune cells, neutrophils seem to be particularly important in cancer-associated thrombosis. Neutrophils represent 50% to 70% of all leukocytes, being the most abundant immune cell population in humans. It has long been demonstrated the presence of adherent leukocytes in the initiation areas of the thrombus [107]. In addition, the histological analyses of human venous thrombi demonstrate the presence of neutrophils, platelets and fibrin layers [107,108]. Studies employing thrombosis models in mice have demonstrated that neutrophils play a major role in the initial steps of thrombus development, being the first cells to accumulate in the injured vessel [109,110]. In this context, neutrophil depletion reduces thrombus formation in a murine flow restriction model of thrombosis [111].

Neutrophil-dependent prothrombotic mechanisms are largely associated with the formation of neutrophil extracellular traps (NETs) [112]. NETs comprise a molecular trap formed by DNA, histones, and several additional proteins including some derived from the neutrophil granules [113]. These structures were first described as a defense mechanism against microbes [114]. It is well-established that NETs are present in both arterial and venous thrombi, either in human or in mice [111,115,116,117]. The importance of NETs on thrombus formation has been proposed based on the antithrombotic effects of molecules that target enzymes required for NETs formation, such as elastase and PAD4 [118,119] or agents that destroy NETs, such as recombinant DNase [111,120].

NETs may induce platelet activation and aggregation, processes that have been associated with histone-dependent and -independent pathways [121,122]. Histone-dependent platelet activation is mediated by toll-like receptors 2 and 4 (TLR2 and TLR4) [123]. Interestingly, activated platelets may promote NETs formation [124,125] thus promoting a vicious cycle that propagates thrombus formation [111]. Platelet-derived high mobility group box 1 (HMGB1) induces NETs formation [126] and impacts venous thrombus formation in mice [127]. In this context, NETs formation was recently associated with the increased platelet aggregation in a murine tumor model [128].

Increased plasma levels of NETs formation markers, such as citrullinated histone H3 (H3cit), cell-free DNA and nucleosomes, have been recently associated with the increased risk of arterial and venous thrombosis in cancer patients [129,130]. Thalin and colleagues showed that patients with advanced cancer present increased levels of H3cit in plasma, correlating with a 2-fold increased risk for short-term mortality [131]. Increased NETs formation markers have been also identified in hepatocellular carcinoma as thrombotic risk factors [132]. In another study, the increased plasma levels of H3Cit and cell-free DNA were associated with higher mortality in patients with cancer but no correlation with arterial thromboembolism has been observed [133]. Tumor-bearing mice also exhibit increased levels of systemic NETs formation. The murine breast cancer model, 4T1, dramatically increase neutrophil counts, NETs formation markers and rely on the establishment of a prothrombotic state [120,134]. Nude mice bearing human pancreatic tumors also exhibit increased venous thrombus formation along with elevation of systemic NETs formation [38]. Remarkably, treatment with recombinant DNase abolishes the prothrombotic state in breast and pancreas tumor models [38,120].

A number of factors may induce NETs formation such as interleukin-8, lipopolysaccharide (LPS), interleukin-1β (IL-1β), G-CSF and others [135,136,137]. In addition, direct exposure of neutrophils to tumor cells [136,138] or tumor cell conditioned medium may elicit NETs formation [139]. G-CSF is a cytokine that has been strongly correlated with cancer-associated neutrophilia [134]. In addition, G-CSF induces NETs release in vitro [136] and in vivo [140]. We have recently demonstrated that tumor-derived EVs induce NETs formation in naïve mice previously treated with G-CSG [120]. In this context, increased G-CSF levels parallel with thrombotic manifestations in human cancer patients [129] and in tumor-bearing mice [38]. Therefore, it is proposed that G-CSF prime the neutrophils towards NETs formation and establishment of several NETs-dependent pro-tumoral effects [141,142].

**Figure 2:**
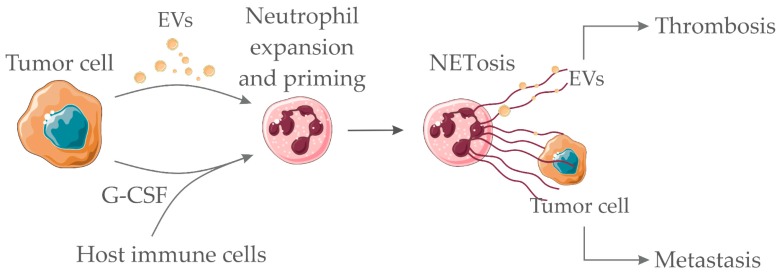
Pro-hemostatic interactions between tumor cell-derived soluble factors/EVs and neutrophils. Tumor-derived EVs and/or tumor/host-derived soluble factors (such as granulocyte colony-stimulating factor (G-CSF) and other cytokines) interact with neutrophils promoting the neutrophil extracellular trap (NET) formation process (NETosis). NETs act as scaffolds for the binding of procoagulant tumor-derived EVs, therefore, amplifying thrombus formation. NETs may also entrap tumor cells, thus favoring metastasis. Servier Medical Art, https://smart.servier.com/, was used to create this figure, licensed under a Creative Commons Attribution 3.0 Unported License.

The ability of NETs to entrap circulating tumor cells has been proposed as a pro-metastatic effect in different murine tumor models [136,143]. The additional ability of NETs to entrap tumor-derived procoagulant EVs has been observed in murine breast cancer [120] and pancreatic carcinoma [36]. This process may contribute to amplifying the establishment of prothrombotic states. Figure 2 depicts the proposed interaction between soluble factors/EVs derived from tumor cells and neutrophils.

## 5. Polyphosphate-Bearing EVs

In addition to the extrinsic pathway of coagulation, which is initiated upon exposure of TF to blood, fibrin formation might be triggered upon activation of FXII (*F12* gene) into FXIIa by negatively charged surfaces such as those provided by polyphosphate (polyP) [22]. Activation of FXIIa leads to activation of FXI (*F11* gene) into FXIa and further reactions culminate with thrombin formation and fibrinogen cleavage into fibrin. Platelets may secrete procoagulant polyP thus eliciting the contact pathway [144]. PolyP is a linear polymer composed of orthophosphate units that naturally occurs in different length forms varying from a few phosphate units to several thousand. Recently, Nickel and co-workers demonstrated that prostate-cancer derived cells might secrete long-chain polyP into EVs, thus eliciting blood coagulation [145]. Experiments performed with plasma clearly demonstrated that polyP-bearing EVs activate FXII into FXIIa in vitro. Fluorescence microscopy further demonstrated that EVs derived from the prostate cancer cell line, PC3, expose procoagulant polyP forms in their surface. Remarkably, the authors demonstrated that infusion of PC3-derived EVs promote thromboembolism in wild type but not in FXII or FXI knockout mice [145]. Alternatively, blocking of polyP/FXII pathway with a specific antibody, 3F7, provided protection from thromboembolism while not promoting bleeding in mice [146]. Therefore, it has been proposed that targeting the polyP/FXII axis could attenuate cancer-associated thrombosis in determined contexts [147].

## 6. Concluding Remarks

**Figure 3:**
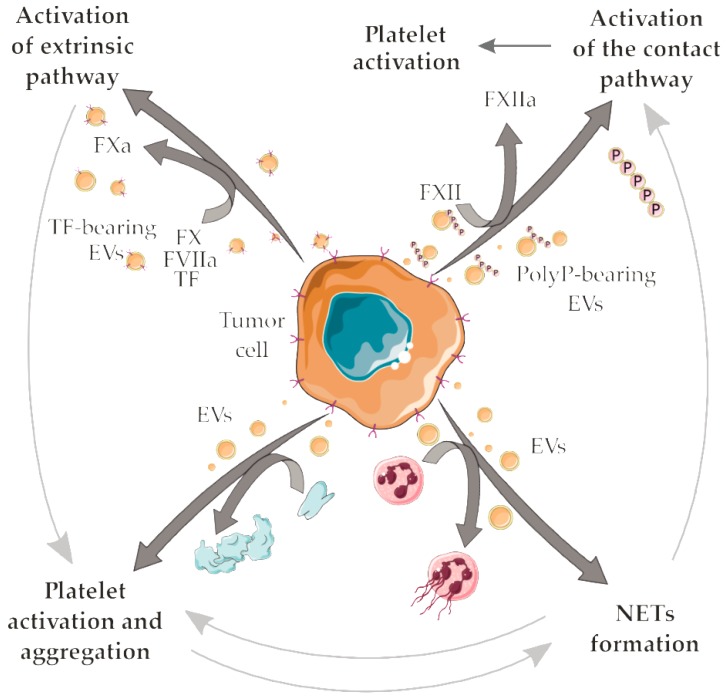
Possible crosstalk between the proposed mechanisms for tumor-derived EVs in cancer-associated thrombosis. Tumor-derived TF + EVs initiate the extrinsic pathway of coagulation. TF is a high-affinity receptor for coagulation factor VII/VIIa (FVII/FVIIa), culminating in the activation of the factors X (FX) into factor Xa (FXa). In turn, FXa mediates the proteolytic conversion of prothrombin to thrombin, a serine protease that amplifies the coagulation cascade and generates fibrin, which stabilizes the platelet plug in clot formation. Further thrombin generation accounts for an indirect mechanism for platelet activation/aggregation. EVs may elicit direct platelet activation/aggregation. Tumor-derived polyP+ EVs initiate the contact pathway of coagulation, mediating the activation of factor XII (FXII) into factor XIIa (FXIIa) by negatively charged surfaces such as those provided by polyP. Further reactions of the contact pathway also culminate with thrombin formation, fibrinogen cleavage into fibrin and platelet activation. Interaction of neutrophils with tumor-derived EVs may support NETs release thus eliciting several NETs-dependent prothrombotic mechanisms. NETs provide additional surfaces that support the contact pathway activation. In addition, crosstalk between platelets and NETs may play an important role in the establishment of cancer-associated thrombosis. Servier Medical Art, https://smart.servier.com/, was used to create this figure, licensed under a Creative Commons Attribution 3.0 Unported License.

A number of prothrombotic mechanisms have been proposed to explain cancer-associated thrombosis. Some of these mechanisms rely on complex interactions between tumor-derived EVs and vascular cells or with components of the hemostatic system. Initiation of the extrinsic pathway of coagulation by TF+ EVs or initiation of the contact pathway by polyP+ EVs may predominate in certain cancer types (Figure 3). In addition, the interaction of tumor-derived EVs with platelets (promoting platelet activation and/or platelet aggregation) or neutrophils (facilitating NETs release) may provide additional positive feedback for the establishment of prothrombotic states (Figure 3). The compilation of the EVs molecules with prothrombotic potential is shown in Table 2. The complexity of the pathogenesis of cancer-associated thrombosis is nicely demonstrated there: one or more mechanisms may take place in a certain cancer type, but not all the proposed mechanisms will simultaneously account for the establishment of the prothrombotic state. This scenario certainly difficult the prediction of thrombotic manifestations, as well as therapeutic interventions. In this context, strategies that could attenuate the crosstalk between platelets and NETs formation [148,149] could potentially reduce cancer-associated thrombosis. Additional crosstalk between the mechanisms herein discussed may also reveal potential targets for intervention (Figure 3).

## Figures and Tables

**Table 1:** Clinical studies associating tissue factor (TF)-containing extracellular vesicles and venous thromboembolism.

Cancer Type	TF Measurement	VTE
Pancreatic, non–small cell lung, ovarian, colorectal and breast [15,41,42]	TF antigen (IFC)	Yes
Colon, lung, bladder, pancreatic, prostate, rectal, bile duct, brain, cholangio, liver, lymphoma, renal cell, testis and other types of cancer [15,41,42]	TF activity (FXa generation assay)	Yes
Gastrointestinal, lung, pancreatic, prostatic, breast, liver, uterine and brain [15,41,42]	TF antigen (FACS)	Yes
Pancreatic [43,44,45]	TF activity (FXa generation assay), TF antigen (FACS or ELISA)	Yes
Breast [43,46]	TF activity (FXa generation assay), TF antigen (FACS)	Yes/ No
Soft tissue sarcoma [47]	TF antigen (FACS)	No
Non-Hodgkin lymphoma, colorectal, breast, stomach, lung and pancreatic [48]	TF antigen (ELISA), TF activity (FXa generation assay)	No
Multiple myelomas [49]	TF activity (FXa generation assay)	No
Ovarian [50,51]	TF antigen (ELISA), TF activity (FXa generation assay or FGT)	No
Small cell lung cancer [52]	TF activity (FXa generation assay)	No
Gastric, colorectal and brain [53]	TF activity (FXa generation assay)	No

TF, tissue factor; IFC, Impedance-based flow cytometry; FACS, fluorescence-activated cell sorting; ELISA, enzyme-linked immunosorbent assay; FGT, fibrin generation test.

**Table 2:** EVs-derived molecules and their possible prothrombotic roles.

EVs-Linked Molecules	Suggested Effect
TF	Activation of the extrinsic pathway, fibrin generation, and platelet activation/aggregation in a thrombin-dependent manner
Podoplanin	Platelet aggregation
PSGL-1	Accumulation of the EVs at the site of thrombosis through binding to platelets via P-selectin
Integrins	Accumulation of the EVs at the site of thrombosis through binding to platelets
Unknown	Neutrophil activation and NETs release
Unknown	Binding to NETs
PolyP	Activation of the contact pathway, fibrin generation, and platelet activation/aggregation in a thrombin-dependent manner

EVs, extracellular vesicles; TF, tissue factor; PSGL-1, P-selectin glycoprotein ligand-1; PolyP, polyphosphate; NETs, neutrophil extracellular traps.
